# Supplementary material for: Inequity in access to personalized medicine in France: Evidences from analysis of geo variations in the access to molecular profiling among advanced non-small-cell lung cancer patients: Results from the IFCT Biomarkers France Study
Source: PLoS One. 2020 Jul 1;15(7):e0234387. doi: 10.1371/journal.pone.0234387 (PMC7329126; doi:10.1371/journal.pone.0234387)
Supplement: S1 Appendix — (DOCX) [file pone.0234387.s001.docx]

**Appendix 1: Summary of our data source**

| Data | Source |
| --- | --- |
| Counts of all demand of tests for advanced non-small lung cell cancers in 2012/2013 | IFCT, BIOMARQUEURS France study. |
| Counts for all first admissions for lung cancer | ATIH, PMSI-MCO 2013 |
| Socio-economic variables (population data to compute densities, poverty rate and proportion of patients having CMUC) | - SAE 2013, traitements DREES ; INSEE, estimations de la population au 1er janvier 2013. + Revenus Disponibles Localisés, Insee-DGI |
| Departmental per capita supplies of health workforce | SNIIRAM datamarts, 2012*  **SNIIRAM data that we use is mostly aggregated data for active health professionals. Individual data on health supply is collected and shared via “datamarts” available online.* Source to better understand SNIIRAM data: Présentation du SNIIRAM, January 2015. |
| Departmental per capita supplies of beds and stays (admission rates) | SAE 2013, traitements DREES ; INSEE, estimations de la population au 1er janvier 2013. |
| Presence of a genetic testing center & presence of a referral cancer hospital | The list of genetic centers is given in appendix in INCa reports cited in References. We used those reports to build departmental dummies on the presence of a genetic center/referral cancer hospital. |
