# Supplementary material for: Inequity in access to personalized medicine in France: Evidences from analysis of geo variations in the access to molecular profiling among advanced non-small-cell lung cancer patients: Results from the IFCT Biomarkers France Study
Source: PLoS One. 2020 Jul 1;15(7):e0234387. doi: 10.1371/journal.pone.0234387 (PMC7329126; doi:10.1371/journal.pone.0234387)
Supplement: S2 Appendix — Significance levels are the same as reported in Table 2 above. (DOCX) [file pone.0234387.s002.docx]

| **Appendix 2: Spatial regression models to test the stability of our results. Significance levels are the same as reported in Table 2 above.** | | | | |  |
| --- | --- | --- | --- | --- | --- |
|  | (1) | (2) | (3) | (4) | (5) |
| Poverty rate (*dummy w. ref = rate >15%)* | -7.470^**^ (3.732) | -7.436^**^ (3.668) | -7.677^**^ (3.613) | -7.758^**^ (3.572) | -7.541^**^ (3.678) |
| Per capita supply of … |  |  |  |  |  |
| General practitioner (per 100,000) | 0.021 (0.023) | 0.089^**^ (0.039) | 0.086^**^ (0.039) | 0.116^**^ (0.046) | 0.112^**^ (0.049) |
| Surgeons (per 100,000) |  | -2.006^**^ (0.979) | -2.249^**^ (0.978) | -1.721^*^ (1.009) | -1.754^*^ (1.011) |
| Radiotherapists (per 100,000) |  |  | 6.572^*^ (3.803) | 6.196^*^ (3.750) | 6.472^*^ (3.930) |
| Pathologists (per 100,000) |  |  |  | -3.482 (2.461) | -3.395 (2.477) |
| Oncologists (per 100,000) |  |  |  |  | 0.955 (4.083) |
| Beds (per 10,000) | 0.037 (0.094) | 0.060 (0.093) | 0.032 (0.093) | 0.103 (0.104) | 0.099 (0.105) |
| Per-capita admission rate (per 10,000) | -0.540 (0.837) | -1.032 (0.847) | -0.741 (0.850) | -1.125 (0.898) | -1.134 (0.898) |
| Presence of a genetic testing center (dummy) | 0.655 (4.083) | -0.661 (4.082) | 0.523 (4.063) | 2.244 (4.140) | 2.198 (4.141) |
| Presence of a referral cancer hospital (dummy) | -2.707 (4.157) | -0.952 (4.208) | -0.768 (4.134) | -2.276 (4.166) | -2.352 (4.194) |
| Proportion receiving CMUC (per 10,000) | -0.377 (0.507) | -0.387 (0.492) | -0.492 (0.490) | -0.459 (0.488) | -0.476 (0.492) |
| Constant | 57.333^***^ (15.192) | 62.605^***^ (14.924) | 59.345^***^ (14.826) | 60.431^***^ (14.819) | 60.968^***^ (14.950) |
| *Observations* | 93 | 93 | 93 | 93 | 93 |
| *Log Likelihood* | -350.638 | -348.797 | -347.334 | -346.411 | -346.384 |
| *sigma^2^* | 104.772 | 102.530 | 99.040 | 96.038 | 96.105 |
| *Akaike Inf. Crit.* | 721.275 | 719.595 | 718.668 | 718.822 | 720.769 |
| *Wald Test (df = 1)* | 15.004^***^ | 8.663^***^ | 9.693^***^ | 13.538^***^ | 13.065^***^ |
| *LR Test (df = 1)* | 10.822^***^ | 5.858^**^ | 6.427^**^ | 7.970^***^ | 7.413^***^ |
