# Supplementary material for: Inequity in access to personalized medicine in France: Evidences from analysis of geo variations in the access to molecular profiling among advanced non-small-cell lung cancer patients: Results from the IFCT Biomarkers France Study
Source: PLoS One. 2020 Jul 1;15(7):e0234387. doi: 10.1371/journal.pone.0234387 (PMC7329126; doi:10.1371/journal.pone.0234387)
Supplement: S3 Appendix — (DOCX) [file pone.0234387.s003.docx]

**Appendix 3: Moran scatterplots and LISA maps.**

It could be informative to look at the extent of spatial autocorrelation with appropriate mapping technics such as Moran scatterplots and local indicators of spatial autocorrelation. Moran’s scatterplots enable to better visualize the magnitude of Moran Index as well as to decompose the spatial autocorrelation [21] [22]. The scatterplot for genetic testing rates is represented below. Each point represents a ‘département’: The rates are on the X-axis and their spatial lags on the Y-axis. Rates have been standardized and given in standard deviational units (mean is zero and standard-deviation is one). The slope is the value of the global Moran Index. On the upper right and lower left of the map, we have the two areas which capture cases of positive spatial autocorrelation, while on the lower right and upper left of the map, we have the two areas of negative spatial autocorrelation.


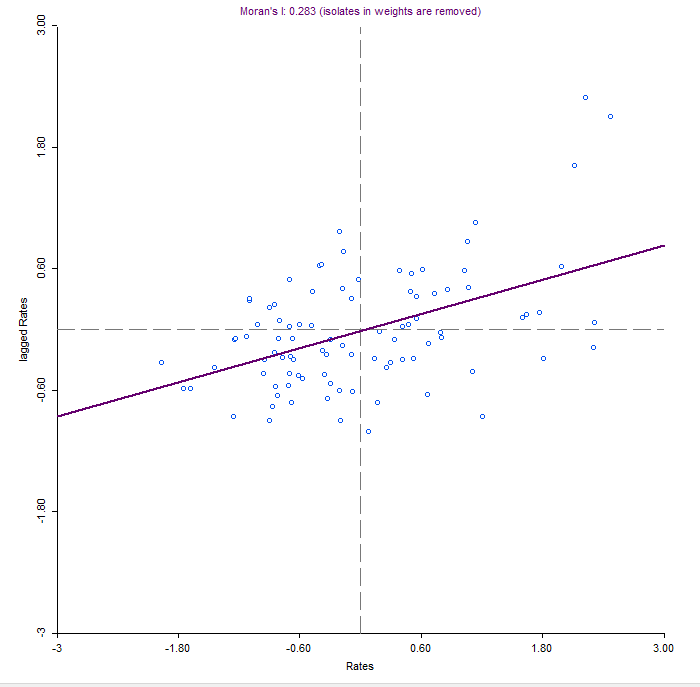


**Figure 6: Moran’s scatterplot for genetic testing rates for NSCLC in France among inhabitants aged 20-99 (left) and those aged 60-99 (right), April 2012 – April 2013.**

Apart from the univariate Moran scatterplot which shows the magnitude of the global spatial autocorrelation indicator (Moran’s I), we can also assess the local significance of the patterns observed on a map representing geographical rates (Figure 1 in the paper). We can therefore identify local significant clusters and challenge the assumption of homogeneity behind the global Moran’s I. Local Indicators of Spatial Association LISA [23] are useful tools to refine the analysis of spatial autocorrelation. LISA will help detect whether there are local clusters and compute local Moran’s I for each spatial unit and evaluate the statistical significance for each local Moran’s I. Figure 7 below shows our 93 local indicators of spatial autocorrelation (computations are performed in Geoda, which uses the Local Moran’s I proposed by Anselin L., 1995 [23]). We have 12 départements where high rates (low rates) cluster with high rates (low rates). The map also enables the visualization of the 7 clusters where there is spatial dispersion of rates (low-high and high-low départements). Given that our main interest is not in discovering the “unexpected” in terms of geographical clusters, but rather in analyzing the eventual association between testing rates and the economic status of the “département”, we have rather focused on robust regression analysis of this relationship, with the advantage that multivariate regression analysis allows us to account for potential confounding factors that also affect the geographical patterns.


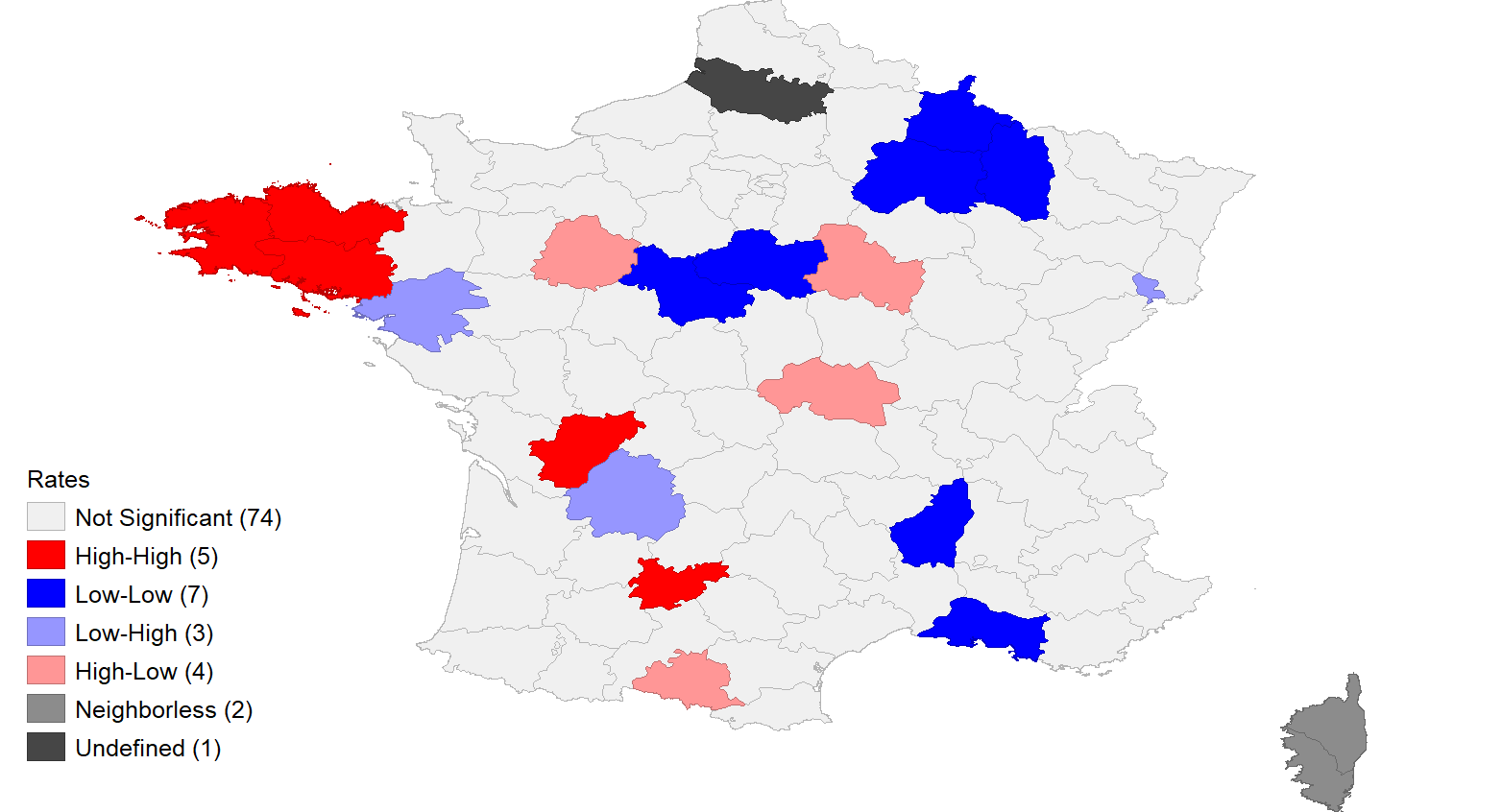


**Figure 7: LISA scatterplot for genetic testing rates for NSCLC in France among inhabitants aged 20-99 (left) and those aged 60-99 (right), April 2012 – April 2013.**
