# Supplementary figures and images for: Inequity in access to personalized medicine in France: Evidences from analysis of geo variations in the access to molecular profiling among advanced non-small-cell lung cancer patients: Results from the IFCT Biomarkers France Study
Source: PLoS One. 2020 Jul 1;15(7):e0234387. doi: 10.1371/journal.pone.0234387 (PMC7329126; doi:10.1371/journal.pone.0234387)

**Appendix 4: Numbered map of French départements**


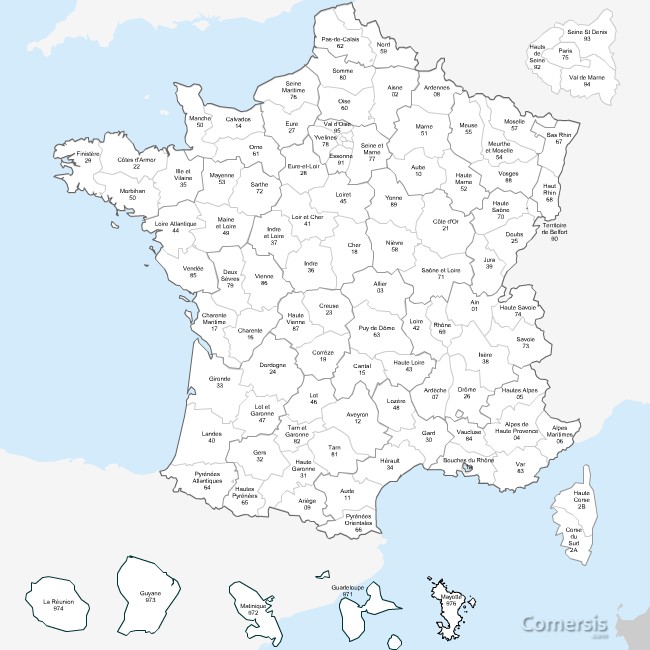

Supplement: S4 Appendix — (DOCX) [file pone.0234387.s004.docx]
