# Supplementary material for: Inequity in access to personalized medicine in France: Evidences from analysis of geo variations in the access to molecular profiling among advanced non-small-cell lung cancer patients: Results from the IFCT Biomarkers France Study
Source: PLoS One. 2020 Jul 1;15(7):e0234387. doi: 10.1371/journal.pone.0234387 (PMC7329126; doi:10.1371/journal.pone.0234387)
Supplement: S5 Appendix — Rates of genetic testing rates for NSCLC in France among inhabitants aged 20–99 (left) and those aged 60–99 (right), April 2012 –April 2013. (DOCX) [file pone.0234387.s005.docx]

**Appendix 5: Rates of genetic testing rates for NSCLC in France among inhabitants aged 20-99 (left) and those aged 60-99 (right), April 2012 – April 2013.**

| **NAME OF THE DÉPARTEMENT** | **CODE ON THE MAP** | **RATE ENTIRE SAMPLE** | **RATE OLDER SAMPLE** |
| --- | --- | --- | --- |
| AIN | 01 | 50.60 | 47.26 |
| AISNE | 02 | 37.56 | 34.93 |
| ALLIER | 03 | 62.08 | 57.65 |
| ALPES-DE-HAUTE-PROVENCE | 04 | 43.89 | 37.94 |
| ALPES-MARITIMES | 06 | 39.54 | 36.60 |
| ARDECHE | 07 | 39.27 | 37.11 |
| ARDENNES | 08 | 32.36 | 28.49 |
| ARIEGE | 09 | 48.44 | 43.49 |
| AUBE | 10 | 38.99 | 40.01 |
| AUDE | 11 | 32.43 | 27.45 |
| AVEYRON | 12 | 66.79 | 54.15 |
| BAS-RHIN | 67 | 55.58 | 49.95 |
| BOUCHES-DU-RHONE | 13 | 45.11 | 39.82 |
| CALVADOS | 14 | 52.50 | 48.74 |
| CANTAL | 15 | 45.32 | 41.35 |
| CHARENTE | 16 | 60.26 | 63.10 |
| CHARENTE-MARITIME | 17 | 53.44 | 45.24 |
| CHER | 18 | 27.17 | 24.39 |
| CORREZE | 19 | 56.31 | 52.66 |
| CORSE-DU-SUD | 2A | n.a | n.a |
| COTE-D'OR | 21 | 40.57 | 35.12 |
| COTES-D'ARMOR | 22 | 77.32 | 74.68 |
| CREUSE | 23 | 34.23 | 28.62 |
| DEUX-SEVRES | 79 | 54.85 | 45.48 |
| DORDOGNE | 24 | 45.42 | 38.98 |
| DOUBS | 25 | 54.14 | 49.89 |
| DROME | 26 | 51.04 | 48.71 |
| ESSONNE | 91 | 57.09 | 54.16 |
| EURE | 27 | 49.21 | 45.13 |
| EURE-ET-LOIR | 28 | 35.24 | 30.96 |
| FINISTERE | 29 | 74.40 | 72.83 |
| GARD | 30 | 30.05 | 28.26 |
| GERS | 32 | 60.44 | 52.97 |
| GIRONDE | 33 | 46.45 | 42.19 |
| HAUTE-CORSE | 2B | n.a | n.a |
| HAUTE-GARONNE | 31 | 47.24 | 40.09 |
| HAUTE-LOIRE | 43 | 38.14 | 34.87 |
| HAUTE-MARNE | 52 | 38.85 | 32.78 |
| HAUTES-ALPES | 05 | 60.90 | 61.15 |
| HAUTE-SAONE | 70 | 53.88 | 45.96 |
| HAUTE-SAVOIE | 74 | 52.56 | 44.81 |
| HAUTES-PYRENEES | 65 | 42.65 | 36.82 |
| HAUTE-VIENNE | 87 | 68.87 | 62.15 |
| HAUT-RHIN | 68 | 75.30 | 66.24 |
| HAUTS-DE-SEINE | 92 | 41.70 | 39.53 |
| HERAULT | 34 | 34.30 | 27.49 |
| ILLE-ET-VILAINE | 35 | 71.51 | 70.99 |
| INDRE | 36 | 33.89 | 30.18 |
| INDRE-ET-LOIRE | 37 | 38.97 | 36.36 |
| ISERE | 38 | 37.68 | 34.71 |
| JURA | 39 | 49.74 | 45.89 |
| LANDES | 40 | 53.61 | 50.80 |
| LOIRE | 42 | 43.01 | 41.28 |
| LOIRE-ATLANTIQUE | 44 | 45.05 | 42.51 |
| LOIRET | 45 | 45.02 | 41.58 |
| LOIR-ET-CHER | 41 | 43.53 | 36.80 |
| LOT | 46 | 59.92 | 53.49 |
| LOT-ET-GARONNE | 47 | 52.16 | 48.56 |
| LOZERE | 48 | 36.05 | 32.67 |
| MAINE-ET-LOIRE | 49 | 39.04 | 36.81 |
| MANCHE | 50 | 54.21 | 49.71 |
| MARNE | 51 | 37.02 | 33.74 |
| MAYENNE | 53 | 41.73 | 38.87 |
| MEURTHE-ET-MOSELLE | 54 | 37.35 | 34.10 |
| MEUSE | 55 | 36.68 | 31.07 |
| MORBIHAN | 56 | 73.07 | 68.64 |
| MOSELLE | 57 | 32.63 | 28.83 |
| NIEVRE | 58 | 23.75 | 21.78 |
| NORD | 59 | 40.08 | 33.40 |
| OISE | 60 | 43.47 | 42.38 |
| ORNE | 61 | 40.20 | 34.65 |
| PAS-DE-CALAIS | 62 | 46.56 | 40.40 |
| PUY-DE-DOME | 63 | 57.16 | 56.91 |
| PYRENEES-ATLANTIQUES | 64 | 57.92 | 54.17 |
| PYRENEES-ORIENTALES | 66 | 26.31 | 23.82 |
| RHONE | 69 | 43.15 | 43.70 |
| SAONE-ET-LOIRE | 71 | 37.19 | 30.33 |
| SARTHE | 72 | 55.50 | 54.07 |
| SAVOIE | 73 | 37.22 | 30.28 |
| SEINE-ET-MARNE | 77 | 45.33 | 42.10 |
| SEINE-MARITIME | 76 | 51.53 | 45.66 |
| SEINE-SAINT-DENIS | 93 | 39.35 | 37.36 |
| SOMME | 80 | n.a | n.a |
| TARN | 81 | 75.38 | 65.94 |
| TARN-ET-GARONNE | 82 | 61.18 | 60.56 |
| TERRITOIRE DE BELFORT | 90 | 42.80 | 38.62 |
| VAL-DE-MARNE | 94 | 69.33 | 61.89 |
| VAL-D'OISE | 95 | 39.08 | 35.12 |
| VAR | 83 | 43.93 | 40.99 |
| VAUCLUSE | 84 | 35.90 | 31.42 |
| VENDEE | 85 | 53.22 | 54.80 |
| VIENNE | 86 | 67.32 | 59.71 |
| VILLE DE PARIS | 75 | 36.60 | 34.46 |
| VOSGES | 88 | 37.85 | 31.85 |
| YONNE | 89 | 49.53 | 46.47 |
| YVELINES | 78 | 46.44 | 41.86 |

This work was approved by French Cooperative Thoracic Intergroup (IFCT-).

Dr. Weeks was funded by the Fulbright Commission and the Institute of Advanced Studies at Aix-Marseille University.
