# Supplementary material for: Inequity in access to personalized medicine in France: Evidences from analysis of geo variations in the access to molecular profiling among advanced non-small-cell lung cancer patients: Results from the IFCT Biomarkers France Study
Source: PLoS One. 2020 Jul 1;15(7):e0234387. doi: 10.1371/journal.pone.0234387 (PMC7329126; doi:10.1371/journal.pone.0234387)
Supplement: Attachment — Submitted filename: response to reviewer.docx [file pone.0234387.s007.docx]

**Inequity in access to personalized medicine in France: Evidences from analysis of small area variations in the access to molecular profiling among advanced non-small-cell lung cancer patients: Results from the IFCT Biomarkers France Study**

Reviewer #1: Review of manuscript PONE-D-19-22289

The manuscript reports on a study conducted in France assessing the access to gene testing for NSCLC lung cancers. It uses geographic analysis and account for availability of health workforce, poverty in the region. The study is overall well conducted and show clear results on the impact of poverty and on lack of access to health workforce as factors explaining the lower access to genetic testing. The use of a large sample of patients, ATIH database (which contain a lot of very under-utilized data for research), and appropriate method is really appreciated.

**General comments:**

1. The text is unclear on whether NSCLC patients included in the study correspond to all patients in France, or only a subset seen in the 28 regional cancer centers. From a quick and dirty check, with around 30000 lung cancer diagnosed per year in France, and with 80% of them being NSCLC, the present study conducted during a year would have captured 70% of all NSCLC in France. This pose the question of transferability of the results and the potential risk of bias. Who are the remaining 30% of NSCLC? They probably live in more remote area, maybe covered by large hospital although not specialized cancer center. And lack of access to a cancer center in the first place could also be associated with poverty, etc… So the present study could have been conducted in a particular subset. If these are indeed 70% of all cancers, the problem is relatively small but still worth mentioning to the reader. If less than 70% it could require further investigation.

Authors:

- We are grateful for this interesting observation. The calculations of the referee have however not accounted for non-squamous and advanced stage NSCLC patients for whom these tests are recommended in priority. A more accurate calculation of the testing rate should have had the departmental population of advanced non squamous NSCLC in the denominator, information that we do not have, but in any case, genetic testing for this subpopulation is way above 45% as shown in earlier studies (see F. Barlesi et al., « Routine molecular profiling of patients with advanced non-small-cell lung cancer: results of a 1-year nationwide programme of the French Cooperative Thoracic Intergroup (IFCT) », The Lancet, vol. 387, no 10026, p. 1415–1426, 2016.).

2. The analysis could also include as parameter the availability of other hospital structure who could provide follow-up of NSCLC.

**Authors:**

- Thank you for this suggestion; in fact, we could think that the availability of other hospital structure (not cancer specialized) could explain bad utilization-rates, especially for poor populations. We included this variable in our estimations, and it was not significant (because the dummy-variable “availability of other hospital structure” has a value “one” for all departments in France).

- Note that the (already included) “Presence of a referral cancer hospital” is a mirror variable in the same spirit. The latter indicates where there is a reference center available for the happy-few; this variable was neither significant.

3. The source of data are not detailed enough. For health workforce density, mentioning SNIIRAM data does not allow to define if the study refers to active workforce, professionally active (active but not always providing clinical care, such as teaching), or licensed to practice. There is also a lack of information on the year of the data used, are these the density for 2019, 2018? For other indicators such as poverty rate etc, the methods are not described, there are several metrics existing to measure poverty, and the source are not clearly identifiable.

Authors:

- Our data indeed comes from a variety of sources (and we believe this is a value-added of the paper, the fact that we cross different source of data to demonstrate and explain geographical differences in use of genetic testing). In the table below, we provide detailed information for our data source. The Table is inserted in the revised manuscript as an appendix. Thanks to the referee’s remark, this value-added has been added in the discussion (first paragraph, second sentence).

*Appendix: Summary of data source*

| Data | Source |
| --- | --- |
| Counts of all demand of tests for advanced non-small lung cell cancers in 2012/2013 | IFCT, BIOMARQUEURS France study. |
| Counts for all first admissions for lung cancer | ATIH, PMSI-MCO 2013 |
| Socio-economic variables (population data to compute densities, poverty rate and proportion of patients having CMUC) | - SAE 2013, traitements DREES ; INSEE, estimations de la population au 1er janvier 2013. + Revenus Disponibles Localisés, Insee-DGI |
| Departmental per capita supplies of health workforce | SNIIRAM datamarts, 2012*  **SNIIRAM data that we use is mostly aggregated data for active health professionals. Individual data on health supply is collected and shared via “datamarts” available online.* Source to better understand SNIIRAM data: Présentation du SNIIRAM, January 2015. |
| Departmental per capita supplies of beds and stays (admission rates) | SAE 2013, traitements DREES ; INSEE, estimations de la population au 1er janvier 2013. |
| Presence of a genetic testing center & presence of a referral cancer hospital | The list of genetic centers is given in appendix in INCa reports cited in References. We used those reports to build departmental dummies on the presence of a genetic center/referral cancer hospital. |

4. The modelling reported on table 2 includes the density of several health care occupations. These are very often highly correlated, and the modelling shows surprising results with some coefficient being positive and other being negative. It would be advisable to check if this is due to collinearity or not. It would be helpful to provide (in an annex) the model with only GP, and then reassure the reader that the progressive addition of other health care workers did not created this phenomenon of coefficient being positive and negative.

- As requested by the referee, we have estimated sequentially the models and we report our regression results below (also added in Appendix in the revised manuscript). There is indeed some correlation in the different variables capturing the healthcare supply. The density of GPs is linked to the densities of other professionals in the department. Depending on the model (from (1) to (5)), the significance of the density of GPs is indeed unstable. We didn’t really focus on that in the paper because this was not of primary interest to us; this variable was mainly used as a control for the density of other cancer-related professions (model (5)). We now propose a more complete table (step by step) that helps to recognize the relative collinearity between variables.

| Spatial regression models | | | | |  |
| --- | --- | --- | --- | --- | --- |
|  | (1) | (2) | (3) | (4) | (5) |
| Poverty rate (*dummy w. ref = rate >15%)* | -7.470^**^ (3.732) | -7.436^**^ (3.668) | -7.677^**^ (3.613) | -7.758^**^ (3.572) | -7.541^**^ (3.678) |
| Per capita supply of … |  |  |  |  |  |
| General practitioner (per 100,000) | 0.021 (0.023) | 0.089^**^ (0.039) | 0.086^**^ (0.039) | 0.116^**^ (0.046) | 0.112^**^ (0.049) |
| Surgeons (per 100,000) |  | -2.006^**^ (0.979) | -2.249^**^ (0.978) | -1.721^*^ (1.009) | -1.754^*^ (1.011) |
| Radiotherapists (per 100,000) |  |  | 6.572^*^ (3.803) | 6.196^*^ (3.750) | 6.472^*^ (3.930) |
| Pathologists (per 100,000) |  |  |  | -3.482 (2.461) | -3.395 (2.477) |
| Oncologists (per 100,000) |  |  |  |  | 0.955 (4.083) |
| Beds (per 10,000) | 0.037 (0.094) | 0.060 (0.093) | 0.032 (0.093) | 0.103 (0.104) | 0.099 (0.105) |
| Per-capita admission rate (per 10,000) | -0.540 (0.837) | -1.032 (0.847) | -0.741 (0.850) | -1.125 (0.898) | -1.134 (0.898) |
| Presence of a genetic testing center (dummy) | 0.655 (4.083) | -0.661 (4.082) | 0.523 (4.063) | 2.244 (4.140) | 2.198 (4.141) |
| Presence of a referral cancer hospital (dummy) | -2.707 (4.157) | -0.952 (4.208) | -0.768 (4.134) | -2.276 (4.166) | -2.352 (4.194) |
| Proportion receiving CMUC (per 10,000) | -0.377 (0.507) | -0.387 (0.492) | -0.492 (0.490) | -0.459 (0.488) | -0.476 (0.492) |
| Constant | 57.333^***^ (15.192) | 62.605^***^ (14.924) | 59.345^***^ (14.826) | 60.431^***^ (14.819) | 60.968^***^ (14.950) |
| *Observations* | 93 | 93 | 93 | 93 | 93 |
| *Log Likelihood* | -350.638 | -348.797 | -347.334 | -346.411 | -346.384 |
| *sigma^2^* | 104.772 | 102.530 | 99.040 | 96.038 | 96.105 |
| *Akaike Inf. Crit.* | 721.275 | 719.595 | 718.668 | 718.822 | 720.769 |
| *Wald Test (df = 1)* | 15.004^***^ | 8.663^***^ | 9.693^***^ | 13.538^***^ | 13.065^***^ |
| *LR Test (df = 1)* | 10.822^***^ | 5.858^**^ | 6.427^**^ | 7.970^***^ | 7.413^***^ |

5. The second limitation (page 11, lines 238-242) suggest in a long parenthesis (remove parenthesis) that it is not an issue, while this could be associated with poverty and as a result with the percentage of genetic testing. References are often misused (for example page 3, line 65, ref 5), or with wrong numbering (for example: page 4, line 101, ref 7). References should be double checked and harmonized.

**Authors:** Thank you, this has been adjusted and we are now cleared in the paper on our hypothesis of independence between poverty rate and the proportion of NSCLC in lung cancer incidence. Our references have also been crosschecked and harmonized.

**Minor comments:**

6. The title of the manuscript mentions small area variation. As the unit of analysis are French regions (departments) and include half a million inhabitants for several of them, it does not match with a label of small area.

**Authors:**

Thank you for this remark. The title has been marginally adjusted to reflect that:

**Inequity in access to personalized medicine in France: Evidences from analysis of ~~small area~~ geo variations in the access to molecular profiling among advanced non-small-cell lung cancer patients: Results from the IFCT Biomarkers France Study**

7. Dartmouth Atlas Project is cited for a method of indirect standardization, as this method is not originally invented for the Dartmouth Atlas Project, but a very classical indirect standardization available in all epidemiology textbook, another reference could be used (if need be). The only addition on the indirect standardization for the reader would be to explain in two sentences how it is computed and the notion that no population data are required.

**Authors:** Thanks for the remark, a footnote has been added to account for this point (see footnote in Page 5).

8. The method describe the use of a spatial error-lag regression model, this is appropriate but needs a short description of the reason to choose this method for the neophyte.

**Authors:** Thanks for the suggestion. A small description is added in the manuscript (lines 145-146).

9. Add a reference for R software, at least which version was used.

Authors: Ok, thanks. It has been done. Lines 152 – 153

10. Table 1 is unclear: what seems to be reported is the percentage of patients receive genetic testing, while it states “rate of geographical variation”. Same in the footnote, the mention of a rate per 100 advanced NSCLC, again this does not seem to be a rate.
    Table 2, define the metric used (mostly these are estimates from the regression), please also provide the metrics for each variable to enable the interpretation of the results. For example, an estimate of 0.11 for general practitioner corresponds to what? Is it an increase of 0.11% (or 11%) of the percentage of genetic testing for each additional GP per XXX(how many) population?

Authors:

- Thanks for this remark. Table 1 has been renamed appropriately.
- The adjusted rates are over 100. For Table 2, we have added the metric used for each estimation.
- The use of a spatial error model does not change the interpretation: 0.11 for instance captures the slope (assuming linearity as in OLS models) of the rate of genetic testing to the density of general practitioners. This has been added as a footnote in the Table. Lines 210 – 212.

11. In the conclusion line 253-255 page 11, in addition to targeting deprived areas, it would be useful as policy option to recommend reinforcing access to health workforce which implies addressing the challenges of attractiveness and retention in rural/remote areas.

Authors: Thanks for this suggestion, we agree with this point which has been added in lines 298 – 300.

Reviewer #2:

Dear Editors,

The manuscript by Kembou Nzale et al is well written and it is on a very important topic. Ir provides useful inferences and suggestions, which both can help to improve Public Health in France and, if the study s extended to other countries, elsewhere. I suggest minor revisions since the geostatistical analysis of this work could be improved to provide further insights. List of points to be amended:

A. Authors claim that the study was conducted in “mainland France” which “is divided into 94 administrative units called ‘départements”. However, as also shows by the two maps, their study did not include Corse Island, apparently. Authors must clarify this point.

**Authors:**

- We have now clearly stated that Corsica was not part of our sample (we forgot to mention that point in the first version, sorry). We didn’t include it because we didn’t have access to harmonized data for this island. The point has been added between line 104 and 116.

B. The authors employed the Moran Index to assess the extent of spatial autocorrelation of the maps. It would be of interest to insert the Moran scatterplots See: Robertson, Chris, Chiara Mazzetta, and Alberto D’Onofrio. "Regional variation and spatial correlation." Chapter 5 in P. Boyle and M. Smans (Eds.), Atlas of Cancer Mortality in the European Union and the European Economic Area 1993-1997 (2008): 91-113; Anselin L (1995) Local indicators of spatial association Geographical Analysis 27 93–115

**Authors:**

The plot is provided below. It was also added as an appendix in the document. We have added the graph and then the map with the following notes:

*It could be informative to look at the extent of spatial autocorrelation with appropriate mapping technics such as Moran scatterplots and local indicators of spatial autocorrelation. Moran’s scatterplots enable to better visualize the magnitude of Moran Index as well as to decompose the spatial autocorrelation (See: Robertson, Chris, Chiara Mazzetta, and Alberto D’Onofrio. "Regional variation and spatial correlation." Chapter 5 in P. Boyle and M. Smans (Eds.), Atlas of Cancer Mortality in the European Union and the European Economic Area 1993-1997 (2008): 91-113; D'Onofrio, Alberto & Mazzetta, Chiara & Smans, Michel & Boyle, Peter & Boniol, Mathieu. (2016). Maps and atlases of cancer mortality: A review of a useful tool to trigger new questions. Ecancermedicalscience. 10. 670. 10.3332/ecancer.2016.670). The scatterplot is represented below. Each point represents a ‘département’: The rates are on the X-axis and their spatial lags on the Y-axis. Rates have been standardized and given in standard deviational units (mean is zero and standard-deviation is one). The slope is the value of the global Moran Index. On the upper right and lower left of the map, we have the two areas which capture cases of positive spatial autocorrelation, while on the lower right and upper left of the map, we have the two areas of negative spatial autocorrelation.*

Figure 2: Moran scatterplot of the rates of geographic variations

Apart from the univariate Moran scatterplot which shows the magnitude of the global spatial autocorrelation indicator (Moran’s I), we can also assess the local significance of the patterns observed on the map (figure 1 in the paper). We can therefore identify local significant clusters and challenge the assumption of homogeneity behind the global Moran’s I. Local Indicators of Spatial Association (‘LISA’, see Anselin L (1995) Local indicators of spatial association Geographical Analysis 27 93–115) are useful tools to refine the analysis of spatial autocorrelation. LISA will help detect whether there are local clusters and compute local Moran’s I for each spatial unit and evaluate the statistical significance for each local Moran’s I. The Map below show our 93 local indicators of spatial autocorrelation (computations performed in Geoda). We have 12 départements where high rates (low rates) cluster with high rates (low rates). The map also enables the visualization of the 7 clusters where there is spatial dispersion of rates (low-high and high-low départements). Given that our main interest is not in discovering the “unexpected” in terms of geographical clusters, but rather in analyzing the eventual association between geographical rates and the economic status of the “département”, we have focused on robust regression analysis of this relationship, with the advantage that multivariate regression analysis carried out in the analysis allows us to account for potential confounding factors that also affect the geographical patterns observed.

Figure 3: Local indicators of spatial autocorrelation for rates

C. Authors wrote: “autocorrelation was evident (i.e., Moran’s I was <0.001” This is a misprint:, probably the authors refers to the Z--value associated to the Moran I. They must provide the value of the Moran I.

Authors: Moran I statistic is equal to 0.28 with a pseudo p-value < 0.001 for 999 permutations of the model to give test parameters. The misprint is now corrected in the text.

D. The authors provide two maps for two groups of age classes. The maps are visually similar, i.e. they seem highly spatially correlated. However, it would be appropriate to quantify the degree of similarity between them by means of the “two-maps Moran’s scatterplot” and the bivariate Moran’s index, which were both defined in the above mentioned paper by Ronertson et al. See also : d’Onofrio, Alberto, et al. "Maps and atlases of cancer mortality: a review of a useful tool to trigger new questions." ecancermedicalscience 10 (2016).

Authors: Thank you for this suggestion. We have mainly addressed this point from your remark “E” below. In fact, we don’t have two distinct samples, one for “young” and the other for “old”. We rather have an entire sample having all patients aged 1-99, and another subsample where we focus on the 60-99 age group. Given this sample-overlap, we have rather built below the univariate Moran’s scatterplot for the 60-99 to address the referee’s concern in “D”. This map is indeed very similar to the one provided above for the 1-99 age group. This similarity is another visual argument that could justify our choice of splitting the sample to focus on the older age group, the latter representing 72% of lung cancers. Likewise, the value of the Moran Index is approximately the same, so is the LISA map at 5%. We have added these maps in an Appendix of the paper.

Figure: Moran scatterplot for geographic rates in the 60-99 age group.

Figure: LISA map for rates for the 60-99 age group

E. The authors used some spatial data of “ecological” variables: it would be of interest for the reader if the related data would be available. Equally, it would be of extreme interest as an assessment with the univariate Moran index, and (even more interesting) to compare by means of the bivariate Moran index each of these maps with the map of the “rates of use of genetic testing for NSCLC” in the class age 1-59 (and for that in the class 60-99).

**Authors:**

- Our data source has now been inserted in Appendix 1. We have also added a table with all our computed rates for the entire sample and for the older sample (see Table in Appendix 1)
- Our sample is made of the 1-99 population group (entire sample) and we have also focused on the 60-99 age group to better describe what we study.
- Following this remark, two-Moran scatterplots are inserted, and we have shown only cases where associations where significant. LISA maps are also given.

Following the remark, we added this part in the text (after the spatial regressions table) :

*Apart from regression analyses, one could also use spatial analytical tools to visualize the relationships between our computed rates of genetic testing and our ecological variables. We have used the bivariate Moran’s scatterplots as well as the LISA maps to better capture what we describe. We have done this exercise for all our ecological variables and have displayed below only the ones where associations where statistically meaningful. Overall, these maps do not only validate our assumption of spatial correlation between our variables but they also enable us to visualize areas where we have the most significant clusters*.

- We provide in Figure 2 the bivariate Moran’s scatterplot between our computed rates and the poverty rate. The graph confirms the negative association between poverty rates and the rates of genetic testing (already appearing in regression analyses). As displayed on the maps, for the entire sample, the Moran’s I is equal to -0.082 (significant at 5%) and for the subsample of old only, it is equal to -0.11 (significant at 1%). The relationship between poverty rate and computed rates of genetic testing seems stronger for old-age groups.

Figure: Bivariate Moran scatterplot between poverty rate and genetic testing rates

The LISA maps below show significant clusters that drive this relationship. The maps are visually similar for the entire sample and for the older-age group. There are 5 départements in the East where relatively low poverty rates go together with relatively high testing rates. The 4 départements with relatively high poverty rates and low testing rates are not as grouped.

Figure: Bivariate LISA maps between rates of genetic testing and poverty rate (entire sample and subsample of old only).

- The relationship between the density of general practitioners and the computed rates is shown below and it is positive and statistically significant for the two age groups. (the Moran’s I has approximately the same value, significant at 5%).

Figure: Bivariate Moran scatterplot between density of general practitioners and genetic testing rates

- The LISA map shows that there are only 2 départements combining relatively high density of general practitioners and high testing rates. In general, the dominant pattern is rather a relatively low density of general practitioners with relatively lower rates (9 regions for the entire sample map and 7 regions for the subsample of old only).

Figure: Bivariate LISA maps between rates of genetic testing and poverty rate (entire sample and subsample of old only).

F. A practical suggestion: authors ought to include a numbered map of French department, in order that the non French reader can localize the mentioned departments.

Authors: Thank you, this has been done. We have also added it in Appendix and have provided our computed rates for the entire sample and for the older-age group.

Figure: Numbered map of French départements

| **NAME OF THE DÉPARTEMENT** | **CODE ON THE MAP** | **RATE ENTIRE SAMPLE** | **RATE OLDER SAMPLE** |
| --- | --- | --- | --- |
| AIN | 01 | 50.60 | 47.26 |
| AISNE | 02 | 37.56 | 34.93 |
| ALLIER | 03 | 62.08 | 57.65 |
| ALPES-DE-HAUTE-PROVENCE | 04 | 43.89 | 37.94 |
| ALPES-MARITIMES | 06 | 39.54 | 36.60 |
| ARDECHE | 07 | 39.27 | 37.11 |
| ARDENNES | 08 | 32.36 | 28.49 |
| ARIEGE | 09 | 48.44 | 43.49 |
| AUBE | 10 | 38.99 | 40.01 |
| AUDE | 11 | 32.43 | 27.45 |
| AVEYRON | 12 | 66.79 | 54.15 |
| BAS-RHIN | 67 | 55.58 | 49.95 |
| BOUCHES-DU-RHONE | 13 | 45.11 | 39.82 |
| CALVADOS | 14 | 52.50 | 48.74 |
| CANTAL | 15 | 45.32 | 41.35 |
| CHARENTE | 16 | 60.26 | 63.10 |
| CHARENTE-MARITIME | 17 | 53.44 | 45.24 |
| CHER | 18 | 27.17 | 24.39 |
| CORREZE | 19 | 56.31 | 52.66 |
| CORSE-DU-SUD | 2A | n.a | n.a |
| COTE-D'OR | 21 | 40.57 | 35.12 |
| COTES-D'ARMOR | 22 | 77.32 | 74.68 |
| CREUSE | 23 | 34.23 | 28.62 |
| DEUX-SEVRES | 79 | 54.85 | 45.48 |
| DORDOGNE | 24 | 45.42 | 38.98 |
| DOUBS | 25 | 54.14 | 49.89 |
| DROME | 26 | 51.04 | 48.71 |
| ESSONNE | 91 | 57.09 | 54.16 |
| EURE | 27 | 49.21 | 45.13 |
| EURE-ET-LOIR | 28 | 35.24 | 30.96 |
| FINISTERE | 29 | 74.40 | 72.83 |
| GARD | 30 | 30.05 | 28.26 |
| GERS | 32 | 60.44 | 52.97 |
| GIRONDE | 33 | 46.45 | 42.19 |
| HAUTE-CORSE | 2B | n.a | n.a |
| HAUTE-GARONNE | 31 | 47.24 | 40.09 |
| HAUTE-LOIRE | 43 | 38.14 | 34.87 |
| HAUTE-MARNE | 52 | 38.85 | 32.78 |
| HAUTES-ALPES | 05 | 60.90 | 61.15 |
| HAUTE-SAONE | 70 | 53.88 | 45.96 |
| HAUTE-SAVOIE | 74 | 52.56 | 44.81 |
| HAUTES-PYRENEES | 65 | 42.65 | 36.82 |
| HAUTE-VIENNE | 87 | 68.87 | 62.15 |
| HAUT-RHIN | 68 | 75.30 | 66.24 |
| HAUTS-DE-SEINE | 92 | 41.70 | 39.53 |
| HERAULT | 34 | 34.30 | 27.49 |
| ILLE-ET-VILAINE | 35 | 71.51 | 70.99 |
| INDRE | 36 | 33.89 | 30.18 |
| INDRE-ET-LOIRE | 37 | 38.97 | 36.36 |
| ISERE | 38 | 37.68 | 34.71 |
| JURA | 39 | 49.74 | 45.89 |
| LANDES | 40 | 53.61 | 50.80 |
| LOIRE | 42 | 43.01 | 41.28 |
| LOIRE-ATLANTIQUE | 44 | 45.05 | 42.51 |
| LOIRET | 45 | 45.02 | 41.58 |
| LOIR-ET-CHER | 41 | 43.53 | 36.80 |
| LOT | 46 | 59.92 | 53.49 |
| LOT-ET-GARONNE | 47 | 52.16 | 48.56 |
| LOZERE | 48 | 36.05 | 32.67 |
| MAINE-ET-LOIRE | 49 | 39.04 | 36.81 |
| MANCHE | 50 | 54.21 | 49.71 |
| MARNE | 51 | 37.02 | 33.74 |
| MAYENNE | 53 | 41.73 | 38.87 |
| MEURTHE-ET-MOSELLE | 54 | 37.35 | 34.10 |
| MEUSE | 55 | 36.68 | 31.07 |
| MORBIHAN | 56 | 73.07 | 68.64 |
| MOSELLE | 57 | 32.63 | 28.83 |
| NIEVRE | 58 | 23.75 | 21.78 |
| NORD | 59 | 40.08 | 33.40 |
| OISE | 60 | 43.47 | 42.38 |
| ORNE | 61 | 40.20 | 34.65 |
| PAS-DE-CALAIS | 62 | 46.56 | 40.40 |
| PUY-DE-DOME | 63 | 57.16 | 56.91 |
| PYRENEES-ATLANTIQUES | 64 | 57.92 | 54.17 |
| PYRENEES-ORIENTALES | 66 | 26.31 | 23.82 |
| RHONE | 69 | 43.15 | 43.70 |
| SAONE-ET-LOIRE | 71 | 37.19 | 30.33 |
| SARTHE | 72 | 55.50 | 54.07 |
| SAVOIE | 73 | 37.22 | 30.28 |
| SEINE-ET-MARNE | 77 | 45.33 | 42.10 |
| SEINE-MARITIME | 76 | 51.53 | 45.66 |
| SEINE-SAINT-DENIS | 93 | 39.35 | 37.36 |
| SOMME | 80 | n.a | n.a |
| TARN | 81 | 75.38 | 65.94 |
| TARN-ET-GARONNE | 82 | 61.18 | 60.56 |
| TERRITOIRE DE BELFORT | 90 | 42.80 | 38.62 |
| VAL-DE-MARNE | 94 | 69.33 | 61.89 |
| VAL-D'OISE | 95 | 39.08 | 35.12 |
| VAR | 83 | 43.93 | 40.99 |
| VAUCLUSE | 84 | 35.90 | 31.42 |
| VENDEE | 85 | 53.22 | 54.80 |
| VIENNE | 86 | 67.32 | 59.71 |
| VILLE DE PARIS | 75 | 36.60 | 34.46 |
| VOSGES | 88 | 37.85 | 31.85 |
| YONNE | 89 | 49.53 | 46.47 |
| YVELINES | 78 | 46.44 | 41.86 |
